# Clinical Cases

**Published:** 1883-03

**Authors:** E. W. Berridge

**Affiliations:** London


### CLINICAL CASES.

E. W. BERRIDGE, M. D., LONDON.

(1). *Lycopodium in ulcerated throat.* Feb. 11th, 1874. Mrs. H., æt. 28, says she has had ulcerated throat since yesterday at 4 P. M.; the soreness of throat *commenced on right side, extending to left;* to-day there is a whitish ulcer on right tonsil; sharp pain in throat on swallowing, *especially warm drinks;* pains as if bruised all over limbs; frontal headache; brown tongue; pulse 120; yesterday there was shivering. Has taken *Bell.* and *Merc.* in low potencies without result. Ordered inhalations of steam and cold-water compress to throat, and *Lycop.*$^{cm}$ (Fincke) in water every three hours. (This case was treated eight years ago; since then I have found that even such non-medicinal auxiliaries as steam and water are not *necessary*, though they may be used if they give comfort, and no injurious effect.)

Feb. 12th. Slept well; pains in limbs nearly gone; tonsil still ulcerated; pulse 114; tongue light brown; neck externally swelled and tender; free, strong-smelling sweat; mouth dry; urine turbid; no stool; headache, with buzzing in ears and deafness. Continued *Lycopodium.*

Feb. 13th. Feels better; less headache; pulse 90; no pain in limbs; buzzing better; hears better with left ear (has had deafness and buzzing of right ear for many years); ulcer very much better; tongue less furred and less brown; not nearly so much pain on swallowing. Stop medicine.

Feb. 14. Much better; has eaten a mutton-chop; no headache, only giddiness; left ear natural; no pain on swallowing; tongue cleaner.

Feb. 15th. Nearly well; throat healed; only a little pain on swallowing; tongue cleaner; no giddiness. For two days *swelling and redness of tip of nose, with pustules on left side of tip and septum.* (This was an effect of *Lycop.;* I had a similar symptom from a high-potency proving thereof when in health; it shows the value of provings even on the sick.)

Feb. 16th. Came down-stairs yesterday; giddy; appetite good; no pain on swallowing; nose better; tongue cleaner.

Feb. 18th. Yesterday headache and weakness; to-day much better; neck feels stiff; tongue natural; skin of nose, where pustules were, is scaling off (just as it did in my proving).

Feb. 22d. Quite well, except rather weak.

Four of her children had similar attacks, but less severe. The two first attacked were treated by their parents according to one of the works of the late Dr. Ruddock. They *recovered* from the throat symptoms, but were not *cured;* for they were under my care for several days afterward with very large swellings of the parotid glands. The other two, whom I attended from the first, recovered without any *sequelæ.* I have often observed that when the sick are treated mongrel-fashion, or allopathically, though they may recover from the acute symptoms, chronic ailments far more difficult to eradicate are stirred up.

This seldom occurs in the practice of a Hahnemannian, and *never* if he can find a remedy suitable not only to the acute symptoms but also to the constitutional state of the patient.

In the Homœopathic Physician, Vol. II, p. 466, Dr. C. W. Butler* gives, as a frequently verified symptom of *Lycopod.*, "aggravation from cold drinks, except water," and "amelioration from warm drinks." This agrees with the experience of Dr. A. M. Piersons, recorded in the *The Organon*, Vol. II, p. 228. Neither of these physicians seem to consider the aggravation from warm drinks characteristic of *Lycop.*, though it is quoted as being in the materia medica. This proves (1) that clinical symptoms are of extreme importance, and that no repertory or materia medica which excludes them can

* These cases were reported by Dr. J. S. Smith, not by Dr. Butler as credited in our December issue. The error was ours.—Editor.

be exclusively relied on; (2) that a remedy may have two opposite symptoms, one being much more *characteristic* of it than the other; (3) that unless the symptom is characteristic, it requires to be supported by a larger number of other symptoms before we can rely on it in any case; in the above case the direction and time pointed emphatically to *Lycop.* It should also be noticed that the *Lycop.* symptom given by HAHNEMANN is not simply "aggravation of throat from warm drinks," but "if the soup is very warm [*i. e., hot,* not merely warm] he is unable to swallow it."

Will Dr. Gale kindly give me the source of his symptom of *Caust.* (p. 468), "Diplopia, worse on turning eyes to the right"? I have added it to my *Eye Repertory*, which gives only *Digititis.*

The "rare symptom," quoted under *Laurocerasus* (Vol. II, p. 485), is given differently in symptom 12 of Allen's *Encyclopædia.* Is the former from a different proving or a clinical symptom? A proving of *Ether*, which I published in *The Organon*, Vol. II, p. 258, has this symptom.*

(2). *Nitrum in dyspnœa.* 1882, Nov. 15th, Mrs. B. for two days has had catarrh, with feeling as if the lungs were two wooden boards pressing toward the back. One dose of *Nitrum* $^{5m}$ (F. C.) at 9 P. M. Was better in about an hour, and soon cured. *Nitrum* is the only remedy given by Bœnninghausen under "Sensation, as if made of wood," but that *Mat. Med.* does not contain it under "Chest." Another instance of the plan of selecting a remedy by analogy.

(3). *Bromine in headache.* One dose of *Bromine.*$^{cm}$ (Fincke) cured throbbing in left temple; worse on stooping.

(4). *Carbo veg.*$^{cm}$ (Fincke). One dose removed, in fifteen minutes, a pain in left abdomen, as if something were out of place. She had suffered for two hours.

(5). *Magn. carb.* 1881, Sept. 29th, Miss L. had suffered at times since May with pains round left knee, like something tight; only felt at back and front of knee. For the last week the pain has been increasing; left calf hard. To-day there is a hard swelling in hollow of left knee, and she cannot put left foot to the ground when walking, but only when standing. Lippe's *Repertory* (p. 231) gives "Swelling in bend of knee: *Magn. carb.*" On referring to the *Mat. Med.* I found "Hard swelling in hollow of knee; he could not stretch

*Anacardium has anxiety in region of sternum, without pain: feels as if he must go into open air and be busy. This is almost similar to the Lauro. symptom given in volume second, which was from Hering.—EDITOR.

out leg on account of the pain in it." "Pain in bend of left knee on stooping, as if the tendons were too short, etc.," and some other similar symptoms.

I gave one dose of *Magn. carb.*$^{3m}$ (Jenichen) at 8.45 P. M.

Sept. 30th, 9.30 A. M. No swelling or hardness, but stiffness where the swelling was; also stiffness in the corresponding part of right leg. Feeling of tightness decreased.

Nov. 4th. Reports that in about two weeks the symptoms entirely ceased, and there has been no return.

Will any member of the anti-Hahnemannian pathological school explain the pathology of this case, and give a pathological reason for the selection of *Magn. c.*, the only *simillimum* in the whole materia medica? This is only one of the many instances in which Lippe's invaluable *Repertory* has guided me to the remedy. I invariably take it with me to the bedside of the patient, and use it more than any other.

